# Decision-making experiences of family members of older adults with moderate dementia towards community and residential care home services: a grounded theory study protocol

**DOI:** 10.1186/s12877-017-0510-8

**Published:** 2017-06-05

**Authors:** Lisa Pau Le Low, Lai Wah Lam, Kim Pong Fan

**Affiliations:** 1 School of Health Sciences, Caritas Institute of Higher Education, 18, Chui Ling Street, Tseung Kwan O, N.T Hong Kong; 2 Nethersole School of Nursing, Chinese University of Hong Kong, Esther Lee Building, Shatin, N.T Hong Kong

**Keywords:** Community care services, Decision-making, Dementia, Family, Grounded theory, Qualitative interviews, Residential care homes

## Abstract

**Background:**

Caring and supporting older people with dementia have become a major public health priority. Recent reports have also revealed a diminishing number of family carers to provide dementia care in the future. Carers who are engaged in the caring role are known to bear significant psychological, practical and economic challenges as the disease advances over time. Seemingly, evidence indicates that the burden of care can be relieved by formal services. This study aims to explore decision-making experiences of family members of older adults with moderate dementia towards the use of community support (CS) and residential care home (RCH) services.

**Method:**

A large multi-site constructivist grounded theory in a range of non-government organizations and a private aged home will frame this Hong Kong study. Purposive sampling will begin the recruitment of family members, followed by theoretical sampling. It is estimated that more than 100 family members using CS and RCH services will participate in an interview. The process of successive constant comparative analysis will be undertaken.

**Discussion:**

The final product, a theory, will generate an integrated and comprehensive conceptual understanding which will explain the processes associated with decision-making of family members for dementia sufferers. Deeper understanding of issues including, but not exclusive to, service needs, expectations and hopes among family carers for improving service support to serve dementia sufferers in CS and RCH services will also be revealed. Importantly, this study seeks to illustrate the practical and strategic aspects of the theory and how it may be useful to transfer its applicability to various service settings to better support those who deliver formal and informal care to the dementia population.

## Background

Caring and supporting older people with dementia have become a major public health priority regionally and globally [1, 2]. The recent report by the World Health Organization and Alzheimer’s Disease International [1] has highlighted the need to raise awareness of dementia as a public health priority, and to advocate actions to be taken at international and national levels. Together with the recently-released World Alzheimer’s Report [2], the society has been alluded to the latest global and regional trends of older adults who are in great need for dementia care in the long-run, and a global epidemic of a predicted unsustainable shortage of informal carers. This report also alarmingly predicts a three-fold increase in the number of dependent older people, with a figure of 101 million in 2010 to 277 million in 2050, of whom one half of them are living with Alzheimer’s Disease or other form of dementia. In Hong Kong, the current situation among Chinese older people with dementia is escalating, with a prevalence of 1.2% for the 60–65 age group and 32% for the 85-and-above age group [3], suggesting that more care and attention will be needed by those with advancing age. Given the rapid growth in the population aged 65 and over in Hong Kong, of a population of 7 million, 13% are older people aged 65 years and older [4]. It is expected that as older people continue to live longer, the prevalence of those suffering from dementia is expected to increase [5]. The press release by the Hong Kong Alzheimer’s Disease Association [6] has attempted to make explicit headlines of a diminishing number and lack of support for family members. The focus on the need to plan and address both formal and informal care needs for dementia sufferers is urgently warranted [6]. Particularly, this study directs specific attention to informal family caregiving of older people with dementia by taking a retrospective method to examine the decision-making experiences and processes of family members of dementia sufferers towards the use of formal CS and RCH services.

Dementia is a progressive disease of old age characterized by memory loss over time, changes in behaviour, and the degree to which these older people can continue to perform activities of daily living will depend on the severity of the illness. Although caring for family members with dementia can be positive, it is increasingly clear that caregiving can be overwhelmingly cumbersome and a burden to the family caregivers as they are the sole persons in the family to often shoulder the responsibility of care [7]. For example, caregivers are known to bear significant psychological, practical and economics strains as the course of the disease advances [8]. In other studies, caregivers have suffered from sleep disturbance when faced with multilevel stressors [9]. In addition, during the course of the dementia journey there has been a high degree of uncertainty and lack of confidence among families with coping and handling matters that concern their older relatives [10]. Indeed, the plights faced by families and the need to overcome the imperative challenges of being a dementia caregiver are compounded by the general lack of timely and relevant information and appropriate support on dementia and dementia care, as supported by our pilot work that examined decision-making potential and challenges faced by family carers of older people with mild-moderate dementia [11] and by other investigators [12]. Nevertheless, there is a consensus to suggest that the burden of care can be relieved by using formal services (e.g. dementia-specific day care centres [13]) to assist family carers to support people with dementia living in the community, and thereby continue with their care for a longer period [14]. This is in line with the goal of the Hong Kong Government that puts emphasis on the family to provide care in the community through the provision of formal services.

As CS and RCH services play a progressively more important role in supporting Chinese older adults with dementia and their family members, it is important to understand the decision-making processes and types of decisions that are made to better support these older people using these services. Although there is a general lack of literature about this phenomenon, studies that make reference to participation, involvement, degree of choice and control in care practices for older people can be used to inform decision-making [15]. In a very recent study, 20 people with dementia or family caregivers were interviewed to explore the decision-making process about living arrangements and future place of care for the older person with dementia. The results showed that during the decision-making process, they will consider several factors, for example, desire to let the older person stay at home in a familiar environment, safe to be left at home, physical health of the older person with dementia and the carer’s health condition [16]. Apart from the health care/medical decision-making that seems to receive attention for this population group, other types of decisions and how they are made by the family in providing the best possible care for these older adults has not yet been explored. Currently, little is known about the respective roles of family members in decision-making for older people with moderate dementia, their level of involvement in decision-making and the influence they have on decisions that determine the dementia sufferers’ lives. This study is an attempt to fill the knowledge gap.

Exploring decision-making of family members of dementia sufferers is an imperative area for investigation in this study to gain deeper understanding of the complex demands and challenges associated with dementia caregiving and how families may then find relieve for themselves and for the dementia sufferers through making decisions to use different types of CS and RCH services. It is also important to understand how the families act as decision-makers for their dementia relatives by examining the processes of how decisions are made to receive the required care to meet their needs. Indeed, the literature about this phenomenon on family decision-making for dementia sufferers and the types of decisions that are related to CS and RCH services are scarce. Therefore, the objectives of this project are:

- ▪ To explore the general decision-making experiences and types of decisions that family members usually need to make with/for older persons with dementia;
- ▪ To identify the extent to which current CS-RCH services have helped family members to meet their own needs and challenges when serving their older relatives with dementia;
- ▪ To explore factors influencing the family members’ decision to decide on CS and/or RCH services for older persons with dementia;
- ▪ To examine experiences and circumstances that have influenced the family to make a decision to continue using the CS-RCH services for older persons with dementia; and
- ▪ To examine the perspectives and roles family members play and the influences they have in shaping the lives of older relatives with dementia when they use the CS-RCH services.

## Methods

### Study design

A two-year constructivist grounded theory (conGT) design will be used [17] to collect the data using semi-structured interviews. This design posits that knowledge is co-created with the participants, and acknowledges them as being the expert. In this case, the family carers become the expert in care, and who are able to share their experiences in the interviews. The inductive approach to managing the data and theory building will unravel co-understanding and interpretations of the multiple meanings surrounding the phenomenon under study.

### Settings and participants

This study will be conducted in CS and RCH services from non-government organizations (NGOs) and a private aged home. The participants are family members of older persons with moderate dementia. Screening older persons for dementia will include a confirmed diagnosis of dementia, and a MMSE score of 11–20 for moderate cognitive impairment. Recruiting family members include those who have an older relative with moderate dementia, are immediate family members (e.g. daughters, sons, spouses or grandchildren), are identified by staff to be the contact person, and are the elder’s primary carer. After the initial purposive sampling of participants, theoretical sampling will follow. In determining the sample size, based on the P.I.’s prior work on decision-making in RCHs that collected 105 qualitative interviews from residents, families and care providers from three residential care homes [18], the guideline used here was to sample approximately 12 family members per site. Therefore, it is estimated that around 100 family members will be recruited from CS and RCH services in order to reach data saturation of the various service types.

### Data collection

The timeframe for this study will be 24 months. Semi-structured individual interviews will be the main data collection method and will be supplemented by field notes. All participants will be interviewed once and will be audio-taped. Examples of broad questions for the family interview were developed. The research assistant (RA) will screen the elders and then recruit family members for the pilot and main study. Pilot interviews will be conducted to revise the interview schedules. For the main study, once an elder with a confirmed diagnosis of dementia has been identified, the person-in-charge and/or their representative will be approached by the RA to identify the elder’s primary caregiver and the suitability for inclusion in the study. The person-in-charge will make the initial point of contact by briefly explaining the study to the identified family member based on the information sheet and consent form. Once verbal agreement to participate in the study has been secured, the family member’s contact details will be given to the RA to arrange a date, time and place for the interview. The interview schedule revolves around a number of topics as follows:

1. Types of care provided to the older person with dementia.
2. Types of decisions made for this older person in the past.
3. Experiences of how the older relative was helped to make those decisions described.
4. Own needs and challenges of being a family caregiver.
5. Process of deciding which CS and/or RCH service to use to best meet the needs of the older person with dementia.
6. Involvement of family carer in making decisions about the services their older relative will need.
7. Types of decisions made for the older relative now that they are using CS-RCH services.
8. Role and influences family carer have made in shaping the older person’s life.
9. Experiences and circumstances that have been influential in continuing to use the CS-RCH services.

### Ethical considerations

Ethical approval to conduct the study was given by the Research and Ethics Committee of Caritas Institute of Higher Education on (3 October 2014 [Ref. no. PW/14/E0008]). Informed written consent will be obtained from the individual participants.

### Data analysis

All the interviews will be transcribed verbatim and its accuracy will be checked by rechecking the scripts against the taped-recordings. Data will then be analyzed using constant comparative analysis methods to generate concepts and to develop the theory through an inductive process of defining, categorizing, comparing data, and explaining and seeking relationships in the data [17]. A systematic approach will be used by the investigators to fully understand the data, compare all the variations in the data, and accounts for the related properties in the categories. ‘MAXQDA The Art of Text Analysis’ will be used to code and categorize the data and later to facilitate data interpretation and report writing.

### Rigor

The quality criteria established to judge grounded theory studies include: ‘credibility’, ‘originality’, ‘resonance’ and ‘usefulness’ in line with Charmaz’s work will be followed [17]. This is a set of evaluative criteria which allow the investigators to explore the broader impact and social relevance of a project.

## Discussion

This 2-year conGT study on decision-making of family carers towards the use of CS & RCH services for older people with moderate dementia will provide pointers and deeper conceptual understanding to explain the processes and the experiences family members have been through (if in a RCH) or are still at the heart of providing care to their loved ones (if using CS). The final product, the theory, gives an impression that this is all about an academic and impractical endeavour. On the contrary, the resultant theory is highly practical and places emphasis on ensuring the concepts that emerge are closely grounded in the participants’ data [19]; that is, the theory encompasses the stories of our family members who are the experts in caring. However, it needs to be noted that the phenomenon ‘decision-making’ is an abstract concept to the laymen. Particularly, in this study, some family members of our older adults are themselves in their old age and may encounter difficulty in their understanding of the term. Therefore, inviting them to provide illustration of events and perspectives of decision-making that they have undertaken will ensure that the data obtained will have greater meaning.

Deeper understanding of issues including, but not exclusive to, service needs, expectations and hopes among family carers for improving service support to serve dementia sufferers in CS and RCH services will be revealed. Indeed, care that is provided by a spouse to the older person with dementia can be entirely different from that provided to a parent, particularly when the offspring’s gender is different from the parent they care for. Therefore, a larger sample size from various sites is planned in this study. Despite this, data collection will stop when data saturation is reached. An anticipated constraint posed upon sampling may be the difficulty of getting a balanced number of male to female carers, especially when female carers have known to dominate. As participants are recruited from multiple sites which will enhance the transferability of the study, the constant comparative analysis method and theoretical sampling will facilitate the investigators to identify the most appropriate participants to interview and thus enhance the credibility of the study and the identification of the decision-making process towards the use of CS and RCH services.

There are two limitations identified. First, since participants are interviewed once, details and subtle changes in the decision-making process have to be based on the participants’ ability to recall incidents that have happened at different points of time. Second, only participants who have identified themselves as the primary caregivers of the older persons with dementia are interviewed. However, some of them may not be the principal decision-makers for the older adults’ major welfare, particularly when these caregivers are financially dependent on other family members. For future studies, it is recommended to interview the primary decision-makers for the older persons’ major welfare in addition to the older person’s other primary caregiver(s) if they are of different persons.

As stated at the outset of this paper, currently there is a lack of support for family caregivers of older persons with dementia. After understanding caregivers’ caring experiences and their decision-making process, interventions can be planned to support these caregivers. If caregivers are better supported, the need for CS or RCH services can be deferred to a later stage. Indeed, dementia is a chronic, progressive and irreversible condition. Once caregivers have the feeling of burnout, they may totally leave the care of their older relative in the hands of professional care staff such as opting to transit into RCHs. Consequently, supporting caregivers may facilitate them to continue their care for the older relatives even after they are using formal CS or RCH services. Ultimately, this study will seek to illustrate the practical and strategic aspects of the theory and how it may be useful to transfer its applicability to various service settings to better support those who deliver formal and informal care to the dementia population.

## Abbreviations

CS: Community support
RA: Research assistant
RCH: Residential care home
